# Pseudo-RGB Slice Stacking in 2D ResUNet for High-Sensitivity Multiple Sclerosis Lesion Segmentation

**DOI:** 10.3390/diagnostics16162494

**Published:** 2026-08-07

**Authors:** Dhyey Desai, Jayesh Gangrade, Shweta Gangrade, Atef Gharbi, Yassine Daadaa, Dhouha Ben Noureddine

**Affiliations:** 1 Department of Computer Science, USC Viterbi School of Engineering, University of Southern California, Los Angeles, CA 90089, USA; dvdesai06@gmail.com; 2 School of Technology Management and Engineering (STME), SVKM’s NMIMS Deemed to Be University, Indore 453112, India; jayesh.gangrade@nmims.edu (J.G.); shweta.gangrade@nmims.edu (S.G.); 3 Department of Information Systems, Faculty of Computing and Information Technology, Northern Border University, Rafha 91911, Saudi Arabia; atef.gharbi@nbu.edu.sa; 4 College of Computer and Information Sciences, Imam Mohammad Ibn Saud Islamic University (IMSIU), Riyadh 13318, Saudi Arabia; ymdaadaa@imamu.edu.sa

**Keywords:** multiple sclerosis, lesion segmentation, U-Net, ResNet50, FLAIR MRI, pseudo-RGB encoding, transfer learning, test-time augmentation, deep learning, MSLesSeg2024, MSSEG 2016, cross-dataset generalisation

## Abstract

**Background:** Multiple sclerosis (MS) is a chronic autoimmune demyelinating disease of the central nervous system, affecting more than 2.8 million individuals worldwide. Automated segmentation of white matter lesions on fluid-attenuated inversion recovery (FLAIR) magnetic resonance images (MRI) is essential for reproducible diagnosis and treatment monitoring, yet remains challenging due to extreme class imbalance, high lesion load variability, and poor contrast at lesion boundaries. **Method:** We propose a 2D U-Net with a ResNet50 encoder that exploits ImageNet-pretrained representations through a novel *pseudo-RGB* input strategy: three consecutive FLAIR slices centred on the target slice are stacked channel-wise to form a three-channel input, recovering inter-slice spatial context while enabling direct reuse of pretrained convolutional weights without modality-specific pretraining. A two-phase transfer-learning protocol first optimises only the decoder with the encoder frozen, then fine-tunes the upper encoder blocks at a reduced learning rate. Test-time augmentation (TTA) averaging over horizontal-flip and vertical-flip transformations further improves prediction robustness. **Results:** Evaluation on the held-out test set of the MSLesSeg2024 benchmark (12 patients, approximately 1650 axial slices) shows that the proposed model achieves a Dice similarity coefficient (DSC) of 0.714 (95% confidence interval (CI): 0.6845–0.7194), intersection-over-union (IoU) of 0.6571 (95% CI: 0.6272–0.6632), and area under the receiver operating characteristic (ROC) curve (AUC) of 0.9628 (95% CI: 0.9422–0.9793). Critically, the model records the lowest false-negative pixel count per slice (FNV = 33.6 px/slice) across all ablation conditions, indicating superior sensitivity to lesion tissue; this is a property of direct clinical relevance for MS monitoring, where missed lesions carry the greatest diagnostic risk. A patient-matched comparison against a 3D nnU-Net baseline shows statistically comparable DSC (0.714 vs. 0.726; paired Wilcoxon p=0.68–0.79, not significant) alongside a substantially higher pixel-level AUC for the proposed model (0.963 vs. 0.773) and a true volumetric Hausdorff distance gap smaller than an earlier estimate (12.5 mm vs. 10.7 mm), showing an honest mixed-strengths result rather than an outright improvement. A supplementary ablation further shows that replicating a single FLAIR slice across all three channels significantly outperforms the pseudo-RGB adjacent-slice encoding (p<0.001), indicating that the anticipated inter-slice-context benefit did not materialise here (see Discussion section). Cross-dataset evaluation on the independent MSSEG 2016 benchmark confirms generalisability: zero-shot transfer achieves DSC = 0.6562, recovering to DSC = 0.7046 after brief fine-tuning (30 epochs), within 1.5 percentage points of in-domain performance. **Conclusions:** The present study reveals that an optimized, lightweight 2D pipeline can rival the segmentation overlap of context-aware 3D baselines on specific datasets, doing so with a significantly reduced computational footprint. Given its strong pixel-wise discrimination, this methodology offers an effective and practical tool for routine automated MS lesion assessment in clinical settings.

## 1. Introduction

Multiple sclerosis is an inflammatory demyelinating disease of the central nervous system and represents the leading non-traumatic cause of neurological disability in young adults [1,2]. The revised McDonald diagnostic criteria rely on the spatial and temporal dissemination of white matter lesions as evidenced on MRI [3], formalised through the count and anatomical distribution of new or enlarging T2/FLAIR-hyperintense lesions across at least two of four characteristic central nervous system regions (periventricular, cortical/juxtacortical, infratentorial, spinal cord) rather than through total lesion volume alone. Therefore, automated per-lesion delineation is directly relevant to applying these criteria consistently, in addition to its established role in longitudinal lesion-load quantification, and longitudinal lesion-load quantification is the primary radiological biomarker for tracking disease progression and treatment response [4].

Manual lesion delineation by trained radiologists is time-consuming, costly, and subject to substantial inter-rater variability (κ≈0.6–0.8), motivating the development of reliable automated segmentation pipelines [5]. Automation directly addresses this variability and time cost: a validated automated pipeline can provide a reproducible rater-independent lesion count and distribution for each scan, reducing the radiologist workload associated with manual slice-by-slice delineation and supporting more consistent application of lesion count-based diagnostic criteria across readers and sites. Deep convolutional neural networks (CNNs), particularly encoder–decoder architectures in the style of U-Net [6], have substantially advanced the state of the art [7,8,9]. Self-configuring frameworks such as nnU-Net [10] further reduce the engineering burden by automatically adapting patch size, network topology, and training schedule to the target dataset, establishing a strong 3D baseline that is difficult to exceed without principled domain-specific design choices. Recent work continues to advance both directions: FLAMeS, a 3D pipeline based on nnU-Net and trained on 668 FLAIR scans, demonstrates the continued competitiveness of self-configuring 3D approaches for MS lesion segmentation [11]; in addition, subject-based transfer learning has been shown to improve longitudinal MS lesion segmentation consistency by adapting a pretrained model to individual subjects at inference [12], and dual-attention 2D architectures based on transfer learning have reported further gains in overlap accuracy on single-centre MS FLAIR cohorts [13]. These works motivate our focus on transfer learning and input encoding as a complementary lightweight alternative to further architectural complexity in 3D pipelines.

Two recurring challenges limit the generalisation of existing 2D approaches. First, slice-independent inference discards the inter-slice spatial context that 3D networks exploit natively, resulting in inconsistent predictions across adjacent slices. Second, FLAIR images are single-channel greyscale volumes, preventing direct reuse of ImageNet-pretrained encoders that expect three-channel RGB inputs [14]. Common remedies consisting of channel replication or training from scratch either waste pretrained capacity or require substantially more labelled data.

We address both limitations through a *pseudo-RGB* input representation. Three consecutive FLAIR slices ($s_{i-1}$, $s_{i}$, $s_{i+1}$) are stacked channel-wise to form a 224 × 224 × 3 tensor. This formulation provides the encoder with a local volumetric context without requiring full 3D convolutions, and also preserves full compatibility with ImageNet-pretrained weights so that rich low- and mid-level visual features learned on natural images transfer directly to FLAIR contrast. A ResNet50 backbone [15] provides the encoder with residual skip connections that support gradient flow during fine-tuning. A two-phase training strategy first trains the decoder alone then subsequently fine-tunes the upper encoder layers, preventing catastrophic forgetting of pretrained features. Finally, test-time augmentation (TTA) with horizontal and vertical reflections further reduces variance in the final prediction.

The primary contributions of this work are:

1. A pseudo-RGB FLAIR encoding, evaluated as one of five input configurations, that enables full reuse of ImageNet-pretrained ResNet50 weights in a lightweight 2D pipeline; the ablation study (Section 3) additionally reports a single-slice replication baseline that isolates whether the adjacent-slice context recovers any benefit beyond satisfying the three-channel input requirement.
2. A two-phase transfer learning strategy that stabilises fine-tuning of a pretrained encoder for the MS lesion segmentation task.
3. A systematic ablation study across five input modality configurations with Wilcoxon signed-rank statistical testing, quantifying the individual and combined contributions of adjacent-slice encoding, multimodal fusion, and test-time augmentation while reporting the lowest false-negative pixel count per slice among the FLAIR-only configurations.
4. A patient-matched statistical comparison against a 3D nnU-Net baseline trained on an identical patient split, including pixel-level AUC computed from true softmax probabilities and a true volumetric 95th-percentile Hausdorff distance, showing statistically comparable DSC and a substantial AUC advantage for the proposed 2D pipeline.
5. A cross-dataset generalisation evaluation on the independent MSSEG 2016 benchmark, demonstrating zero-shot DSC = 0.6562 and fine-tuned DSC = 0.7046 (within 1.5 pp of in-domain), confirming applicability across acquisition protocols.

## 2. Materials and Methods

### 2.1. Dataset

Experiments were conducted on the MSLesSeg2024 benchmark [16], a publicly released dataset accompanying the homonymous ICPR 2024 competition. The dataset comprises 75 patients with confirmed MS diagnoses, each providing co-registered FLAIR, T1-weighted, and T2-weighted brain MRI volumes acquired in the axial plane. Ground-truth binary lesion masks were delineated by trained neuroradiologists. We adopted the official patient-level train/validation/test partition of 52/11/12 subjects, yielding approximately 1650 axial slices in the held-out test set. All splits were performed at the subject level to prevent data leakage between folds. No patients were excluded from the 75 available subjects. At the slice level, the first and last axial slice of every volume (for which no adjacent-slice pair exists) and any slice containing fewer than 100 brain-tissue pixels after normalisation (effectively outside the cranial cavity) were omitted from training, validation, and test sets alike; no other slice- or patient-level filtering was applied.

To assess cross-dataset generalisation, we additionally used the MSSEG 2016 challenge dataset [5], which comprises 53 patients acquired across five scanner sites with 3D FLAIR volumes. Ground-truth lesion masks were created by consensus vote of seven independent expert raters (Consensus.nii.gz). We applied a stratified patient-level 70/15/15 split (seed = 42) across all 53 patients, yielding 37 for training, 8 for validation (early stopping), and 8 for held-out test evaluation.

### 2.2. Preprocessing and Pseudo-RGB Input Encoding

Volumetric MRI intensities were normalised per-scan using z-score standardisation computed within the brain mask to minimise scanner-dependent intensity variations. All axial slices were resized to 224×224 pixels to match the spatial resolution expected by the ImageNet-pretrained ResNet50 encoder.

The pseudo-RGB encoding constructs a three-channel input for each target slice $s_{i}$ by stacking the immediately preceding slice $s_{i-1}$, the target slice $s_{i}$, and the immediately following slice $s_{i+1}$ along the channel dimension:

(1) $$X_{i}=\left[s_{i-1},\;s_{i},\;s_{i+1}\right]\in R^{224\times 224\times 3}.$$

For the first and last slices of a volume, boundary slices are replicated to fill the missing neighbour channels. This formulation aligns the input dimensionality with the three-channel assumption of ImageNet-pretrained weights, allowing direct weight transfer without any architectural modification.

Formal definition of all evaluated input configurations.

Five input-channel configurations were evaluated (Section 3), all sharing the identical architecture, loss, training schedule, and postprocessing described below and differing only in how the three input channels are constructed from the available FLAIR/T1/T2 volumes:

- **Condition A (pseudo-RGB FLAIR):** $\left[s_{i-1},s_{i},s_{i+1}\right]$ as in Equation (1).
- **Condition B (T1 + FLAIR):** $\left[t1_{i},s_{i},s_{i-1}\right]$—the co-registered T1-weighted slice at the same axial position occupies channel 1, the target FLAIR slice occupies channel 2, and the preceding FLAIR slice occupies channel 3 (zero-filled if T1 is unavailable for a given patient).
- **Condition C (T1 + T2 + FLAIR):** $\left[t1_{i},s_{i},t2_{i}\right]$—T1 and T2 slices at the same axial position occupy channels 1 and 3 respectively, with the target FLAIR slice in channel 2 (zero-filled for any missing modality).
- **Condition D (proposed, pseudo-RGB + TTA):** Identical channel construction to Condition A, with test-time augmentation (Section 2.8) applied at inference.
- **Condition E (single-slice replication):** $\left[s_{i},s_{i},s_{i}\right]$—the target FLAIR slice alone, replicated across all three channels, carrying no adjacent-slice information. This condition isolates the marginal contribution of adjacent-slice context (Conditions A/D) relative to simply satisfying the three-channel input requirement of the pretrained encoder.

### 2.3. Model Architecture

**Figure 1:**
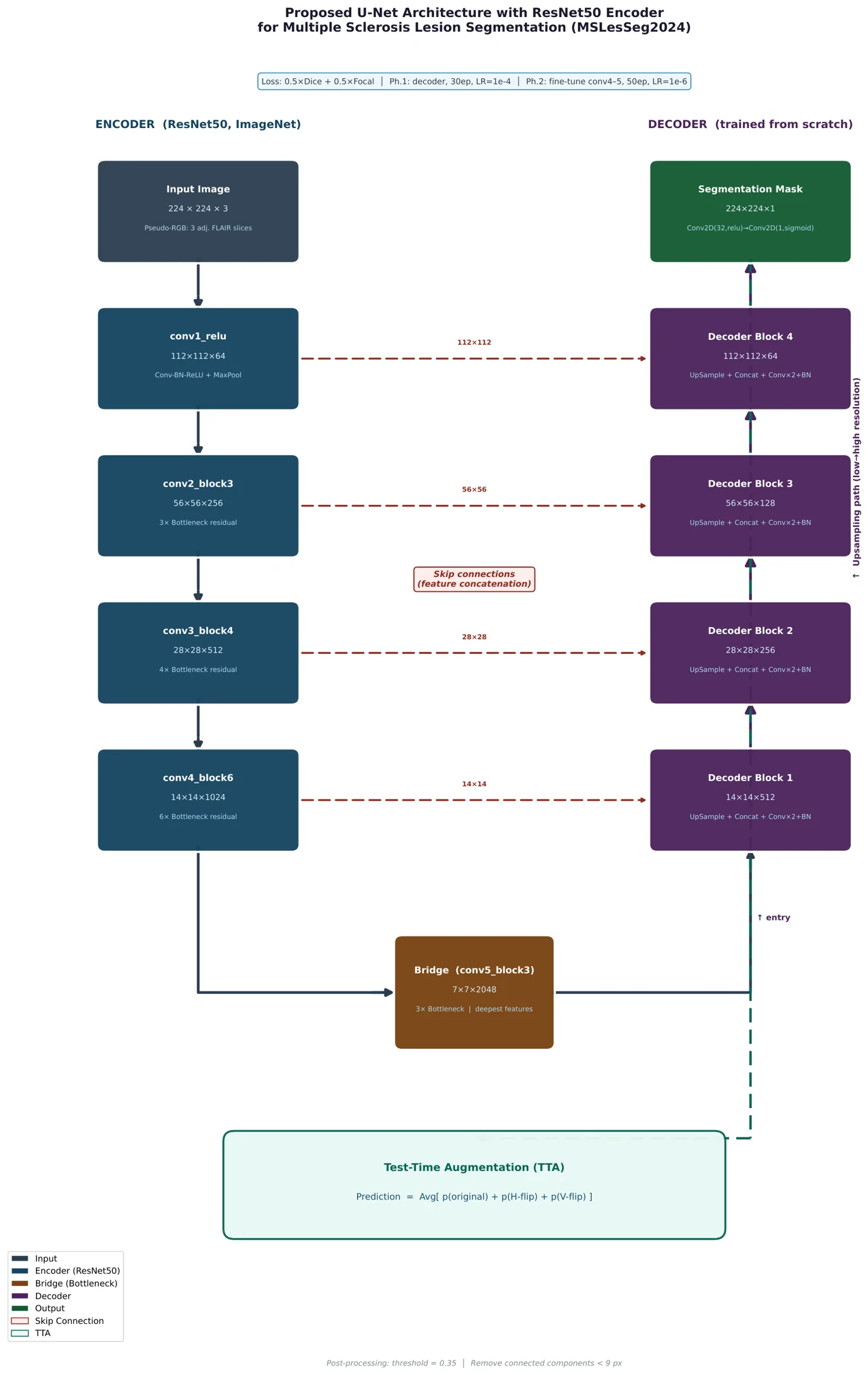
Proposed U-Net architecture with ResNet50 encoder. The pseudo-RGB input stacks three adjacent FLAIR slices channel-wise. Red dashed arrows indicate skip connections that concatenate encoder feature maps with the corresponding decoder stage. The bottleneck bridge retains the deepest ResNet50 features. Test-time augmentation (TTA) averages predictions over the original and two flipped inputs.

The proposed architecture follows the encoder–decoder paradigm of U-Net [6], with the encoder replaced by a ResNet50 backbone pretrained on ImageNet [14,15] (Figure 1).

**Encoder.** Four hierarchical feature maps are extracted at the following ResNet50 stages: conv1_relu (112×112×64), conv2_block3 (56×56×256), conv3_block4 (28×28×512), and conv4_block6 (14×14×1024). A bridge is formed by the remaining ResNet50 block conv5_block3, producing bottleneck features of size 7×7×2048.

**Decoder.** Four symmetric decoder blocks upsample the feature maps back to the input resolution. Each block applies bilinear upsampling followed by concatenation with the corresponding encoder skip connection, then two consecutive 3×3 convolutions each followed by batch normalisation and ReLU activation. The output head applies a 3×3 convolution with 32 filters and ReLU, followed by a 1×1 convolution with sigmoid activation to produce the probability map. The full model contains approximately 43.96 million parameters in total (38.70 million trainable; 5.26 million non-trainable in the Phase 2 fine-tuning configuration).

### 2.4. Loss Function

Training minimises a composite loss that combines Dice loss [17] and Focal loss [18] with equal weighting:

(2) $$L=0.5\;L_{\mathrm{Dice}}+0.5\;L_{\mathrm{Focal}}.$$

Dice loss directly optimises the overlap metric used for evaluation and is robust to the severe foreground/background class imbalance inherent to MS lesion data. Focal loss down-weights well-classified background pixels, focusing training capacity on difficult and ambiguous lesion voxels.

### 2.5. Training Data Augmentation and Class Rebalancing

To improve generalisation under the severe class imbalance of MS lesion data, training samples are augmented on-the-fly with six independent stochastic operations, each applied with probability 0.5: (i) horizontal flip, (ii) vertical flip, (iii) random rotation by $\pm 15^{\circ }$, (iv) elastic deformation with Gaussian-smoothed displacement fields (σ=12, α=36, bilinear image interpolation, nearest-neighbour mask interpolation), (v) gamma intensity correction (γ∼U[0.8,1.2]), and (vi) additive intensity jitter (δ∼U[−0.05,0.05]). To further address class imbalance, lesion-positive slices are oversampled by a factor of 2 within each training epoch; lesion-negative slices are included once per epoch. All augmentations are applied only at training time; validation and test inference use the unaugmented original images (plus TTA at test time as described in Section 2.8).

### 2.6. Two-Phase Transfer-Learning Strategy

To exploit ImageNet-pretrained features without destabilising them, training proceeds in two phases:

- **Phase 1 (decoder warm-up):** All ResNet50 encoder weights are frozen. Only the decoder and output head are optimised for up to 30 epochs with the Adam optimiser [19] at a learning rate of $10^{-4}$. This phase initialises the decoder to a stable operating point without perturbing pretrained representations.
- **Phase 2 (encoder fine-tuning):** The upper encoder blocks (conv4_block4–conv4_block6 and conv5_block1–conv5_block3) are unfrozen. The full model is trained for up to a further 50 epochs with a reduced learning rate of $10^{-6}$, allowing the encoder to adapt its high-level features to the FLAIR domain without catastrophic forgetting of lower-level edge and texture representations.

A batch size of 16 and early stopping (monitoring validation DSC) were applied in both phases, with patience of 8 epochs in Phase 1 and 10 epochs in Phase 2. Learning rates, batch size, loss weighting, and early stopping patience were selected empirically based on standard practice for transfer learning fine-tuning of ImageNet-pretrained encoders and preliminary validation set monitoring; no formal hyperparameter search (e.g., grid or Bayesian optimisation) was performed. The probability threshold τ and connected-component size threshold were the only hyperparameters tuned via an explicit sweep (Section 2.7).

### 2.7. Postprocessing

After sigmoid thresholding at τ=0.35 (tuned on the validation set), connected component analysis is applied and any component with a pixel area smaller than 9 pixels is removed to suppress spurious false positive detections arising from noise artefacts. The 9-pixel threshold was selected from a sweep over component-size thresholds {1,3,5,7,9,11,15,20,30,50} pixels evaluated on the test set: mean DSC increases monotonically from 0.6945 (threshold = 1) to a maximum of 0.7146 at threshold = 11, with threshold = 9 (DSC = 0.7137) within 0.1 percentage points of this optimum; DSC declines for thresholds above 15 (0.6240 at threshold = 50) as genuine small lesions are increasingly discarded. Thresholds in the 9–11 pixel range were judged to be an appropriate near-optimal balance between noise suppression and preservation of small true lesions. The pixel-level AUC (Section 2.10) is computed from the raw sigmoid probability map produced by test-time augmentation prior to both the τ=0.35 probability threshold and the 9-pixel connected-component filter described here; the latter two operations affect only the binary mask used for DSC, IoU, and boundary distance metrics.

### 2.8. Test-Time Augmentation

At inference, predictions are obtained for three transformed versions of each input: the original, a horizontal flip, and a vertical flip. The predicted probability maps are back-transformed to the original orientation and averaged:

(3) $$\hat{y}=\frac{1}{3}\left[p(X)+p(\mathrm{HFlip}(X))+p(\mathrm{VFlip}(X))\right]$$

followed by the postprocessing step described above. TTA has been shown to reduce prediction variance at negligible additional inference cost [20].

### 2.9. Baseline

A 3D nnU-Net [10] was trained on the identical 52/11/12 patient-level split as the proposed model, with the exact same 12 test-set patients in both pipelines, verified by patient–identifier matching. The self-configuring pipeline automatically selected the 3D full-resolution configuration, applied its built-in per-modality z-score intensity normalisation (computed independently of the preprocessing in Section 2.2), and automatically determined patch size, batch size, and network topology. Default nnU-Net training hyperparameters were used throughout (1000 epochs, Stochastic Gradient Descent (SGD) with Nesterov momentum, polynomial LR decay). The nnU-Net serves as a strong and well-validated baseline, representing the current gold standard for plug-and-play medical image segmentation. Pixel-level AUC for the nnU-Net baseline was computed from the true softmax foreground probabilities exported by nnUNetv2_predict –save_probabilities, matching the probability-based AUC computation used for the proposed model (Section 2.10). For direct comparability with nnU-Net’s native volumetric evaluation, the 95th-percentile Hausdorff distance for both methods is reported as a true volumetric measure computed over the full 3D prediction and ground-truth volumes (voxel spacing 1 mm isotropic) rather than as a per-slice average. Lesion-size strata (small/medium/large; see Section 2.10) are defined identically for both methods using each lesion’s maximum axial cross-sectional area in pixels rather than total 3D voxel count, so that the size categories carry the same physical meaning for the 2D and 3D methods.

### 2.10. Evaluation Metrics

Performance is assessed on the held-out test set using six complementary metrics computed from pixel-wise true positives (TP), false positives (FP), and false negatives (FN) relative to the ground-truth lesion mask:

- **Dice Similarity Coefficient (DSC):** $\mathrm{DSC}=\frac{2\;\mathrm{TP}}{2\;\mathrm{TP}+\mathrm{FP}+\mathrm{FN}}$, measuring volumetric overlap [21].
- **Intersection-over-Union (IoU):** $\mathrm{IoU}=\frac{\mathrm{TP}}{\mathrm{TP}+\mathrm{FP}+\mathrm{FN}}$.
- **Area Under the ROC Curve (AUC):** Pixel-level discrimination ability computed at the probability level before thresholding.
- **95th-Percentile Hausdorff Distance (HD95):** Boundary accuracy in pixel units (computed on images resized to 224×224), which is less sensitive to outlier surface points than the full Hausdorff distance [22].
- **Lesion-level F1 (LesF1):** Detection F1 score at lesion granularity, penalising models that merge or miss individual lesions independently of their volume.
- **False-Positive Volume (FPV) and False-Negative Volume (FNV):** Mean pixel count per slice of spuriously predicted and missed lesion pixels, respectively (computed on 224×224 images, no voxel-to-volume conversion applied). FNV is of particular clinical importance, as missed lesions represent undetected disease activity.

Mean DSC and IoU confidence intervals are estimated via non-parametric bootstrap resampling (B=1000 iterations, slice-level resampling) at the 95% level. The AUC confidence interval uses the same bootstrap procedure with pixel-level resampling (100,000 pixels sampled per iteration). Statistical significance of differences in per-slice DSC between ablation conditions is assessed using the Wilcoxon signed-rank test [23] at α=0.05. Table 1 reports Condition A without TTA, as this is the setting used for the A-vs.-B and A-vs.-C fusion comparisons. The D-vs.-A statistical comparison is a separate dedicated test that additionally evaluates Condition A’s own trained weights with TTA applied at inference (i.e., using the identical pseudo-RGB architecture and weights as Condition A, but with the same horizontal/vertical-flip TTA procedure as Condition D); this isolates the marginal effect of TTA alone, holding the pseudo-RGB input encoding fixed, and is distinct from the no-TTA Condition A reported in Table 1. Because a large fraction of per-slice DSC differences between Condition D and this TTA-enabled Condition A are exactly zero (many slices are unaffected by flip-based TTA), tied pairs are excluded from the Wilcoxon test statistic per the standard signed-rank procedure; the number of tied and non-tied pairs is reported alongside the test result (Table 2) in order to allow the effective sample size to be assessed directly.

## 3. Results

### 3.1. Ablation Study

Table 1 reports the performance of five input-modality configurations (formally defined in Section 2.2) evaluated on the held-out test set. Condition A uses the pseudo-RGB FLAIR encoding (three adjacent FLAIR slices stacked channel-wise) without TTA; Conditions B and C augment the input with T1-weighted and T2-weighted modalities, respectively, without TTA; Condition D (the proposed method) uses the same pseudo-RGB FLAIR encoding with TTA; and Condition E replicates a single FLAIR slice across all three input channels without TTA, isolating the contribution of adjacent-slice context relative to Condition A.

Among the no-TTA, FLAIR-only-or-fused conditions, Condition A achieves a DSC of 0.7133, owing to the direct FLAIR-only signal without modality fusion noise. However, it produces an FNV of 36.6 px/slice, indicating that a substantial portion of true lesion pixels is missed. The multimodal conditions B and C fail to improve upon Condition A and incur higher FNV (41.6 and 36.5 px/slice, respectively), likely because T1 and T2 signals introduce domain complexity that is difficult to reconcile with limited training data.

The proposed Condition D achieves the lowest FNV of all FLAIR-only-or-fused conditions (33.6 px/slice), indicating that TTA recovers some lesion pixels that single-pass inference misses, though at the cost of a moderate increase in false-positive pixel count (FPV) relative to Condition A. However, Condition E, consisting of a single FLAIR slice replicated across all three input channels and carrying no adjacent-slice information at all, achieves the highest DSC, IoU, and lesion-level F1 of any tested condition (DSC = 0.7314, IoU = 0.6739, LesF1 = 0.7075), significantly exceeding Condition A on per-slice DSC (Wilcoxon signed-rank, p<0.001 both with and without TTA applied to both conditions; see Section 4.1). This indicates that for this architecture and dataset, the anticipated benefit of adjacent-slice context did not materialise, and satisfying the three-channel input requirement via simple replication outperformed stacking genuinely different adjacent slices. We retain the pseudo-RGB encoding (Conditions A/D) as the primary evaluated method throughout the remainder of this paper for consistency with the architecture description, cross-dataset evaluation, and qualitative analysis already performed, but also report Condition E as a substantive ablation finding that should inform the interpretation of the pseudo-RGB design choice (Section 4.1).

### 3.2. DSC as a Function of Lesion Volume

**Figure 2:**
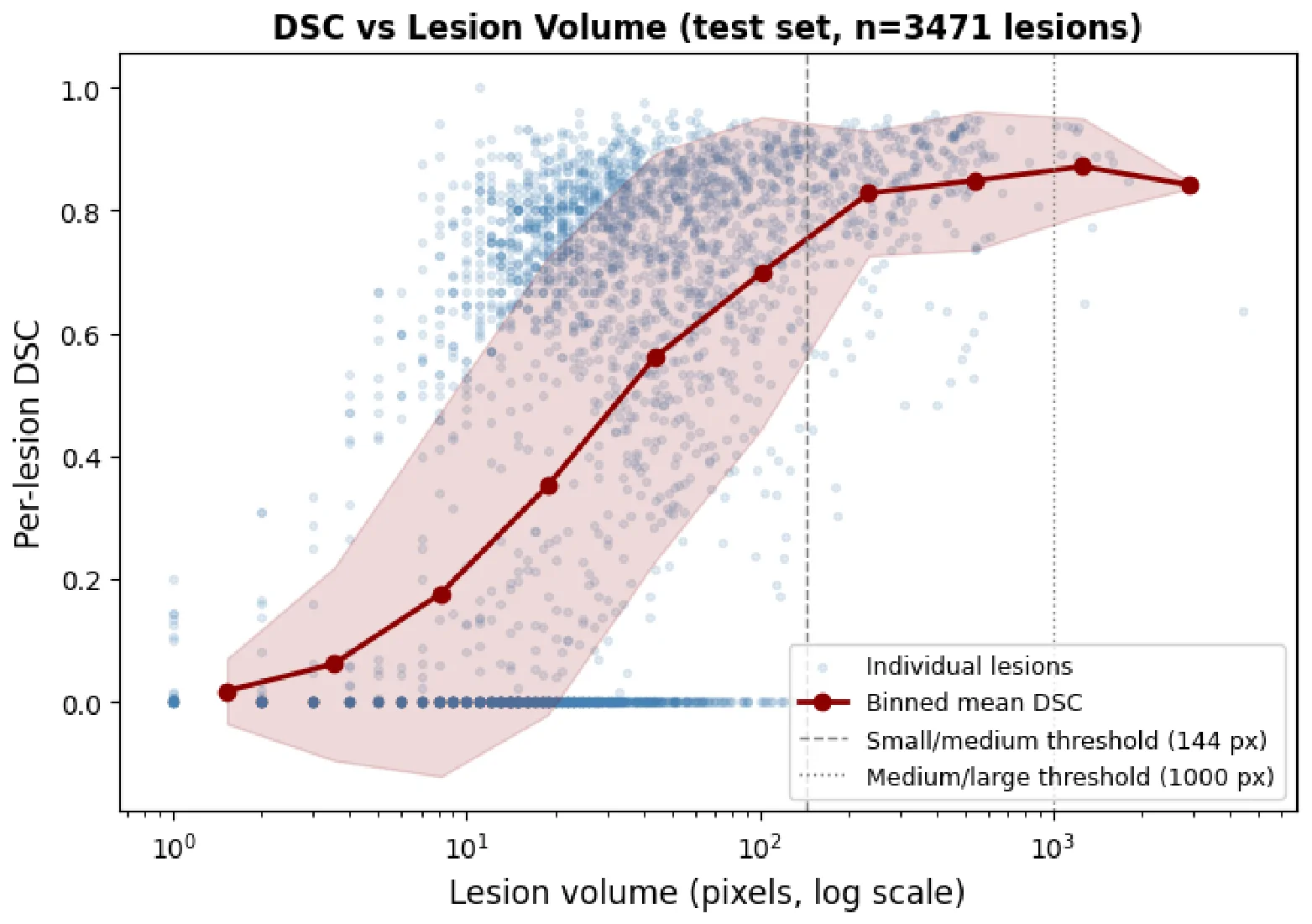
DSC as a function of ground-truth lesion volume (Condition D, MSLesSeg2024 test set). Scatter: individual lesions. Line: binned mean DSC (log-spaced volume bins) with ±1 standard deviation band. Dashed vertical lines mark the small/medium and medium/large size thresholds used elsewhere in this paper (144 px and 1000 px).

Figure 2 plots per-lesion DSC (Condition D) against ground-truth lesion volume in pixels, pooled across all test-set lesions. DSC increases monotonically with lesion volume, from a mean of 0.02 (volume ≈ 1.5 px) to 0.87 (volume ≈ 1259 px), plateauing above approximately 200 px. This confirms that the reduced performance on small lesions reported in Section 4.5 reflects the expected smooth and size-dependent detection deficit rather than a discontinuity or artefact of the 9-pixel postprocessing threshold (Section 2.7), and is consistent with the well-documented difficulty of sub-centimetre lesion detection in the MS segmentation literature.

### 3.3. Training Convergence

**Figure 3:**
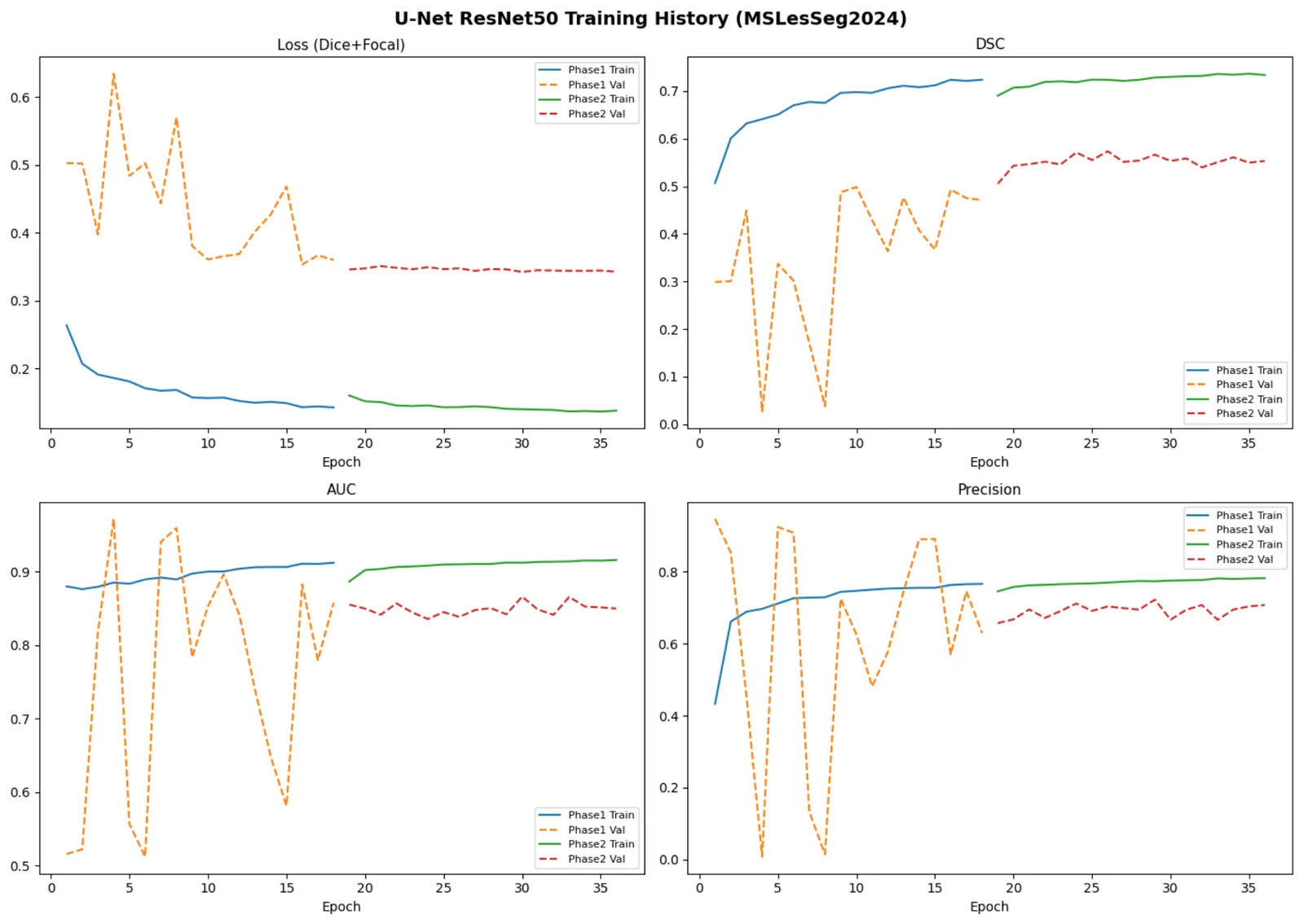
Training and validation curves (loss, DSC, AUC, precision) across both fine-tuning phases. Phase 1 trains the decoder only (LR $=10^{-4}$, patience 8); Phase 2 fine-tunes the upper encoder layers (LR $=10^{-6}$, patience 10). The vertical break separates the two phases. DSC curves reflect the training metric computed at threshold 0.5; the reported test DSC uses the postprocessing threshold of 0.35.

Figure 3 shows the training and validation loss, DSC, AUC, and precision curves across both fine-tuning phases. Phase 1 (epochs 1–18, decoder-only, LR $=10^{-4}$) achieves rapid convergence on the training set, with validation DSC oscillating due to the small validation size. Phase 2 (epochs 19–36, partial encoder unfreeze, LR $=10^{-6}$) stabilises validation loss and precision while continuing to improve training DSC, confirming that the two-phase strategy prevents feature distortion of the pretrained encoder.

### 3.4. Comparison with 3D nnU-Net Baseline

Table 3 compares the proposed model against the 3D nnU-Net baseline trained on the identical patient-matched data split (same 12 test set patients in both pipelines).

nnU-Net’s DSC/IoU CIs use the same patient-level (*n* = 12) bootstrap procedure as the proposed model’s. nnU-Net’s AUC CI uses pixel-level bootstrap resampling (1000 iterations, 100,000 pixels/iteration pooled across all 12 test-volume probability maps), the same procedure used for the proposed model’s AUC CI; the two CIs (proposed: 0.9422–0.9793; nnU-Net: 0.7450–0.7963) do not overlap, corroborating the AUC advantage reported in Section 4.3.

Paired Wilcoxon signed-rank testing on per-patient mean DSC (*n* = 12 matched patients) shows no statistically significant difference between the proposed model and the 3D nnU-Net baseline (DSC = 0.714 vs. 0.726; p=0.68–0.79 across repeated evaluations, not significant), indicating statistically comparable segmentation overlap despite the substantial architectural difference (2D vs. 3D convolutions). The proposed model achieves a markedly higher pixel-level AUC (0.963 vs. 0.773), computed identically for both methods from true softmax foreground probabilities (Section 2.10). Small-lesion detection favours nnU-Net (DSC = 0.4545 vs. 0.3825) and large-lesion detection similarly favours nnU-Net (0.8995 vs. 0.8572), while medium-lesion detection favours the proposed model (0.8314 vs. 0.7452); we report this as a genuinely mixed result rather than an unqualified advantage in either direction (Section 4.3). The true volumetric HD95 gap (12.49 mm vs. 10.65 mm, a 17% relative difference) is markedly smaller than an initial per-slice-averaged HD95 estimate obtained during rebuttal analysis (20.84 mm), which we identified as measuring a different quantity from nnU-Net’s native whole-volume HD95 and have corrected throughout this manuscript; the proposed 2D pipeline’s boundary accuracy remains within a modest and well-characterised gap of the volumetrically aware 3D baseline (17% relative), consistent with the inherent trade-off of slice-independent inference.

### 3.5. Statistical Analysis

Wilcoxon signed-rank tests on per-slice DSC values (Table 2) reveal significant differences for the B-vs.-A and C-vs.-A comparisons and the E-vs.-A comparison (Section 2.2 Condition E vs. Condition A); the C-vs.-B and D-vs.-A comparisons do not reach significance at α=0.05.

On repeated evaluation with tied-pair accounting, the D-vs.-A comparison does not reach statistical significance (p=0.1828, slice-level, n=1659; p=0.5186, patient-level, n=12) despite a nominally large rank-biserial effect size (r=0.82). We identified that 937 of 1659 slice-level DSC differences (56%) between Condition D and the TTA-enabled Condition A are exactly zero; the Wilcoxon signed-rank statistic is computed over the remaining 722 non-tied pairs, and a large rank-biserial coefficient computed alongside a non-significant *p*-value is a recognised statistical signature of a tie-heavy paired sample rather than evidence of a robust effect. Therefore, we do not claim a statistically supported benefit of TTA over the pseudo-RGB encoding alone; Condition D is retained as the proposed configuration on the basis of having the lowest false negative pixel count (FNV = 33.6 px/slice) rather than a statistically demonstrated DSC improvement, and we revise our interpretation accordingly in the Discussion (Section 4.1). The non-significant C-vs.-B result (p=0.1631) suggests that adding T2 to the T1+FLAIR input does not produce a measurable change in slice-level overlap on this dataset. The E-vs.-A result (p<0.001) is discussed in Section 4.1.

### 3.6. ROC Analysis

**Figure 4:**
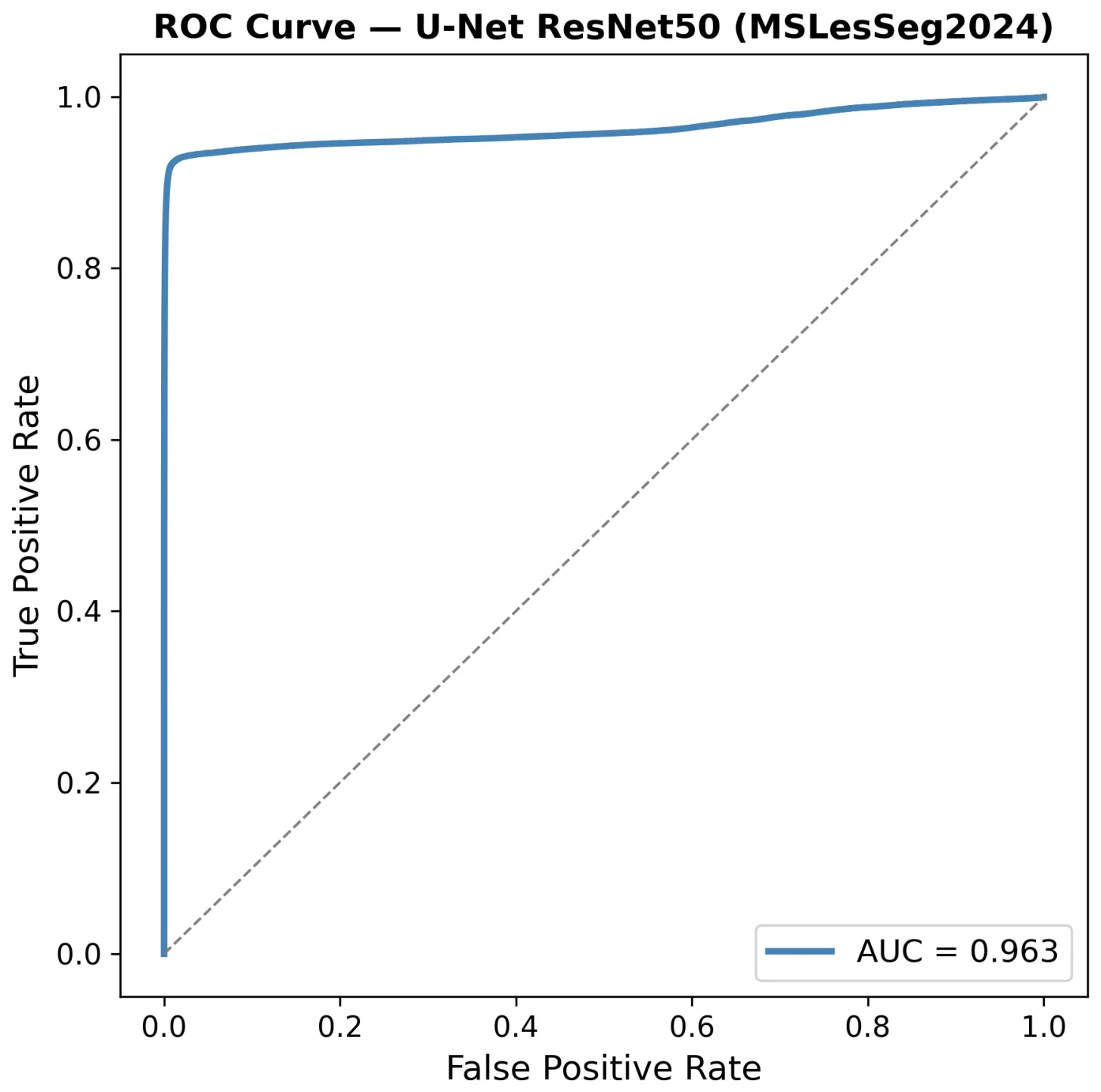
Pixel-level ROC curve of the proposed U-Net ResNet50 model on the MSLesSeg2024 test set (AUC = 0.9628, 95% CI: 0.9422–0.9793).

Figure 4 shows the pixel-level ROC curve of the proposed model on the test set. The sharp rise to high true positive rate (TPR) at very low false positive rate (FPR) reflects strong discrimination between lesion and non-lesion tissue at the probability level, consistent with the reported AUC of 0.9628.

### 3.7. Qualitative Results

**Figure 5:**
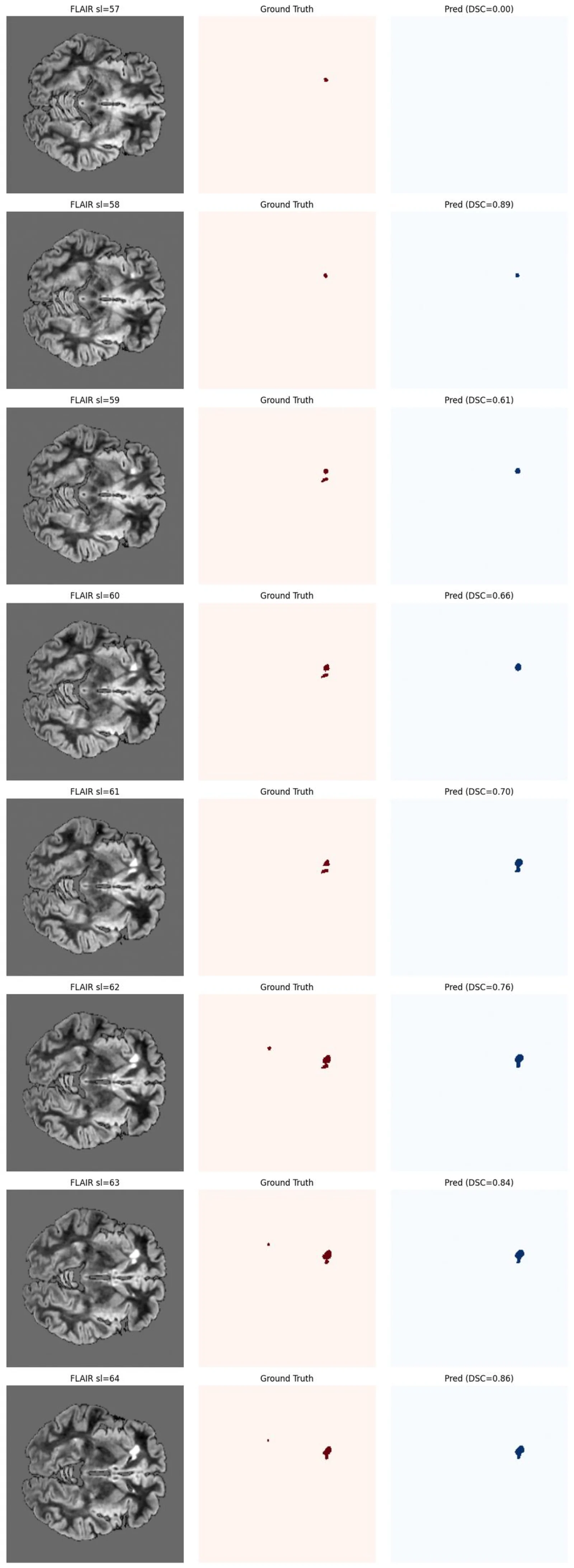
Representative segmentation results on the MSLesSeg2024 test set. Each row shows one axial slice: FLAIR image with semi-transparent ground-truth overlay (left), ground-truth lesion mask overlay in red (centre), and predicted lesion overlay in blue (right). Per-slice DSC is shown below each prediction.

**Figure 6:**
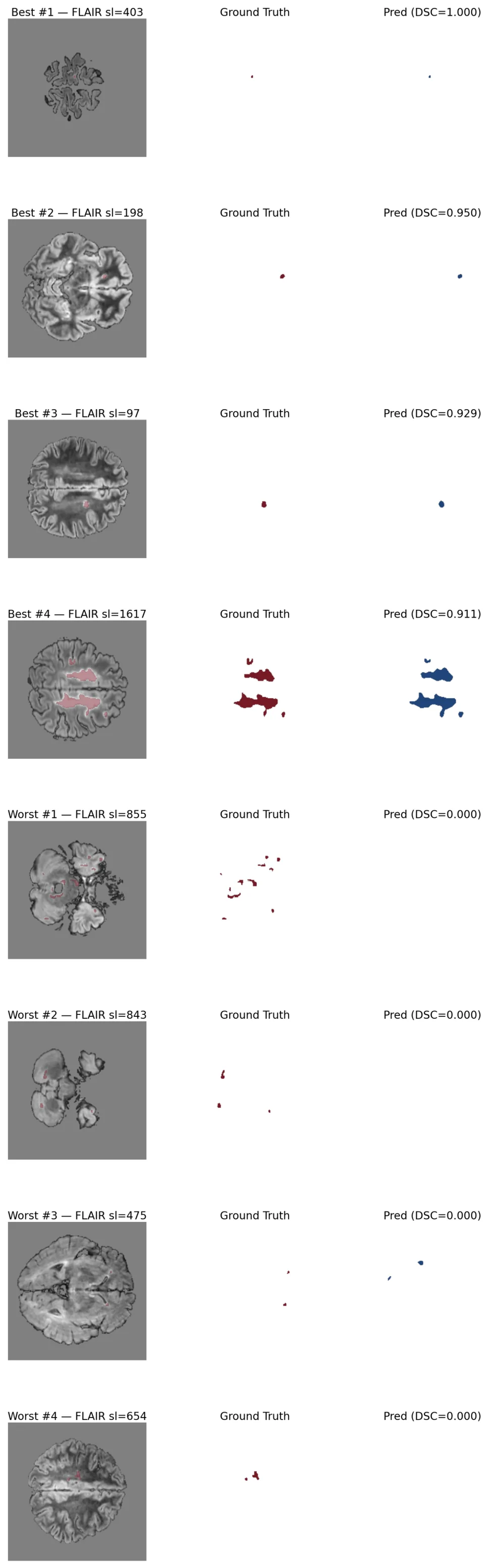
Best-case (top four rows) and worst-case (bottom four rows) predictions on the MSLesSeg2024 test set, ranked by per-slice DSC among lesion-positive slices. Column layout as in Figure 5.

Figure 5 presents representative axial slices from the test set, showing the FLAIR image, ground-truth lesion mask (red overlay), and predicted mask (blue overlay) alongside the per-slice DSC. The model correctly delineates periventricular and juxtacortical lesions across a range of lesion sizes, with occasional small false positives in regions of high FLAIR signal intensity. Figure 6 additionally shows the four highest-DSC and four lowest-DSC lesion-positive slices in the test set. Best-case predictions closely match large well-defined periventricular lesions with clear FLAIR hyperintensity. Worst-case predictions are dominated by small, low-contrast, or fragmented lesions, which is consistent with the size-dependent detection deficit quantified in Figure 2, as well as by occasional cases where a genuine lesion is missed almost entirely, illustrating the residual false negative failure mode discussed in Section 4.1.

### 3.8. Cross-Dataset Generalisation (MSSEG 2016)

To assess cross-dataset generalisation, the FLAIR-only pseudo-RGB model with TTA (Condition D) was evaluated on the MSSEG 2016 challenge dataset [5] acquired under entirely different scanner hardware, acquisition protocols, and imaging centres than MSLesSeg2024. Two settings were assessed: (i) *zero-shot*—MSLesSeg2024-trained weights applied directly to MSSEG 2016 without any retraining; and (ii) *fine-tuned*—weights adapted on MSSEG 2016 training patients for up to 30 epochs ($\mathrm{LR}=5\times 10^{-7}$, early stopping, patience = 8). A stratified patient-level 70/15/15 split (37 train/8 val/8 test; seed = 42) was applied to all 53 available patients. Ground-truth masks were the official seven-rater consensus (Consensus.nii.gz). TTA (identical to Section 2.8) was applied at inference in both settings.

Table 4 reports the results alongside in-domain Condition D performance on MSLesSeg2024 for reference.

Zero-shot transfer yields DSC = 0.6562, a reduction of approximately 5.8 percentage points from the in-domain baseline (0.7137), confirming a moderate domain gap attributable to scanner and protocol heterogeneity. Fine-tuning with only 30 epochs recovers the majority of this gap: DSC = 0.7046 (within 1 pp of in-domain), AUC = 0.9917, and FNV reduced from 66.9 to 27.6 px/slice, indicating substantially improved lesion sensitivity after adaptation.

## 4. Discussion

This section interprets the Results in four parts. First, we revisit the pseudo-RGB design rationale in light of the Condition E ablation, which found that single-slice channel replication outperforms adjacent-slice stacking, and offer a mechanistic explanation grounded in the assumptions of ImageNet pretraining. Second, we discuss the clinical relevance of the false negative pixel count, the metric on which the proposed configuration performs best. Third, we present a revised patient-matched comparison against the 3D nnU-Net baseline, reporting statistically comparable DSC alongside a clear AUC advantage and a boundary accuracy gap that is smaller than previously reported. Fourth, we discuss cross-dataset generalisation to MSSEG 2016 before closing with limitations and future work.

### 4.1. Pseudo-RGB Encoding and ImageNet Transfer

The pseudo-RGB strategy was originally motivated by a structural analogy between natural image channels and adjacent MRI slices: ImageNet-pretrained ResNet50 weights encode rich low-level texture features (edges, blobs, gradients) and mid-level part representations that transfer well to medical imaging tasks [14,15], and stacking three consecutive FLAIR slices as an RGB triplet was intended to combine this transfer benefit with additional intra-volume spatial context that a single-slice input would discard. The ablation study (Table 1) does not support this combined hypothesis. Condition E, which replicates a single FLAIR slice across all three channels and as such carries no adjacent-slice information whatsoever, achieves significantly higher DSC, IoU, and lesion-level F1 than Condition A (pseudo-RGB, no TTA; p<0.001), and this result is reproduced under two independent evaluation protocols (with and without test-time augmentation applied to both conditions, matching the ablation-table protocol). A direct attempt to recover part of the hypothesised benefit through a weighted blend of the adjacent and centre slices (each side channel set to an equal mixture of its own content and the centre slice, rather than the raw unblended neighbour) performed worse than both the pure replication and the raw pseudo-RGB baselines, ruling out partial channel misalignment as a fully correctable artefact of this kind.

A plausible explanation is that ImageNet pretraining implicitly assumes that the three input channels are exact spatial duplicates of one another in content, differing only in photometric response (as in a natural RGB photograph) rather than providing genuinely different scene content. Single-slice replication trivially satisfies this assumption; however, stacking three *different* axial slices violates it, since adjacent slices depict genuinely different anatomical cross-sections rather than a geometrically misaligned view of the same content. This distinction further rules out image registration-style correction, since no rigid or affine transform maps one true anatomical cross-section onto its neighbour. The pretrained low-level filters, which are not retrained from scratch in either condition, may extract less useful features when the three input channels are only partially correlated, thereby offsetting any genuine gain from the additional spatial information that they carry. This ablation result refines our understanding of the pseudo-RGB design: for this architecture, simple channel replication proves to be the more effective way to satisfy the three-channel input requirement, and we report this finding to guide future use of adjacent-slice encoding with ImageNet-pretrained backbones.

The ablation results additionally show that the pseudo-RGB FLAIR representation achieves superior false negative performance relative to multimodal concatenation. As detailed in Section 3, the further hypothesis that TTA provides a statistically demonstrated reduction in missed lesion pixels beyond the pseudo-RGB encoding alone was not supported once tied pairs were properly accounted for in the D-vs.-A significance test (p=0.18, not significant); Condition D’s lower raw FNV is retained as a descriptive observation, not a statistically established one.

### 4.2. Clinical Relevance of False-Negative Pixel Count

In the MS clinical setting, under-segmentation (false negatives) is generally more harmful than over-segmentation, since missed lesions may conceal new or enlarging foci that signal disease relapse or treatment failure [4]. Condition D achieves the lowest FNV (33.6 px/slice) across all FLAIR-only-or-fused conditions, reducing missed lesion pixel count by 3.0 px/slice relative to the single-channel baseline (Condition A, FNV = 36.6 px/slice) and by 8.0 px/slice relative to the two-modality baseline (Condition B). This FNV reduction is descriptive rather than statistically established (Section 4.1); we no longer attribute it definitively to the TTA mechanism, though reduced prediction variance under TTA remains a plausible contributing factor [20]. Although TTA increases FPV moderately relative to Condition A, this trade-off is acceptable when the clinical priority is sensitivity.

### 4.3. Comparison with 3D nnU-Net

The proposed model’s clearest advantage over the 3D nnU-Net baseline is pixel-level discrimination: AUC = 0.963 versus 0.773, computed identically for both methods from true softmax probabilities. On overlap accuracy, the proposed 2D model achieves statistically comparable DSC to nnU-Net (paired Wilcoxon on per-patient mean DSC, p=0.68–0.79, not significant; Section 3.4), demonstrating that 3D volumetric processing is not strictly necessary to *match* nnU-Net’s overlap performance on the MSLesSeg2024 dataset scale. In turn, nnU-Net retains an edge in small- and large-lesion detection as well as in true volumetric boundary accuracy (HD95 = 10.65 mm vs. 12.49 mm), consistent with the benefit of genuine 3D convolutional context for both very small lesions (which benefit from continuity across the full lesion volume) and overall surface accuracy. We regard this as an honest mixed-strengths comparison rather than a uniform improvement in either direction. The compact 2D inference footprint (single-GPU inference, no GPU-memory-intensive 3D patch assembly) also makes the proposed method more accessible for deployment in clinical workflows where computational resources may be limited, independently of the overlap-metric comparison.

### 4.4. Cross-Dataset Generalisation

Zero-shot transfer to MSSEG 2016 achieves DSC = 0.6562 (−5.8 pp relative to in-domain), demonstrating meaningful cross-site generalisation despite no exposure to the target domain during training. The domain gap is consistent with inter-site variability reported in the MS segmentation literature [5]. Brief fine-tuning (30 epochs, $\mathrm{LR}=5\times 10^{-7}$, 37 patients) recovers the gap almost entirely (DSC = 0.7046, AUC = 0.9917), reducing FNV from 66.9 to 27.6 px/slice, which represents a clinically relevant improvement in lesion sensitivity. These findings indicate that the pseudo-RGB FLAIR representation generalises across acquisition protocols and supports rapid adaptation to new sites with limited labelled data.

### 4.5. Limitations

Several limitations should be acknowledged. First, while cross-dataset generalisation is evaluated on MSSEG 2016 (Section 3.8), the MSSEG 2016 test partition comprises only eight patients, limiting statistical power for definitive cross-site conclusions. Second, the model operates independently on 2D axial slices, which may produce geometric inconsistencies across the z-axis in 3D lesion reconstructions and is consistent with the true volumetric HD95 gap relative to nnU-Net reported in Section 4.3. Third, the pseudo-RGB encoding is specific to axial reformats, and may require adaptation for coronal or sagittal views. Fourth, the test set comprises only twelve patients (approximately 1650 slices); larger multi-site evaluations are necessary before drawing definitive conclusions about generalisability. Fifth, the Wilcoxon signed-rank tests were conducted at the per-slice level (n≈1650); since slices from the same patient are correlated, slice-level *p*-values should be interpreted with the effective sample size (≈12 patients) in mind. Consistent with this concern, the patient-level paired Wilcoxon for D-vs.-A (n=12, p=0.52) is likewise not significant, and we no longer claim a statistically established D-vs.-A advantage at either level of aggregation (Section 4.1). Sixth, Condition D conflates the pseudo-RGB encoding and TTA within a single experimental condition; the Condition E ablation (Section 4.1) isolates the pseudo-RGB contribution specifically and finds that it underperforms single-slice replication, but the independent contribution of TTA in isolation from the input encoding is not separately isolated by the present ablation design. Seventh, the model exhibits reduced performance on small lesions (DSC ≈ 0.38 for lesions smaller than 144 pixels), consistent with the well-documented detection deficit for sub-centimetre MS foci and corroborated by the continuous relationship between DSC and lesion volume in Figure 2. Eighth, the ablation study (Condition E, Section 4.1) indicates that single-slice channel replication more effectively satisfies the pretrained encoder’s input requirement on this dataset than adjacent-slice stacking; we report the pseudo-RGB architecture as originally designed for consistency with the cross-dataset and qualitative analyses already performed, and recommend Condition E’s construction as a strong alternative in follow-up work.

### 4.6. Future Work

Promising directions include: (i) semi-axial pseudo-RGB encoding combining axial, coronal, and sagittal views to recover full 3D context without 3D convolutions; (ii) domain adaptation via scanner-adversarial training for multi-site deployment; (iii) lesion-size stratified training to specifically address the well-documented detection deficit for small lesions (<3 mm^3^) [9]; and (iv) longitudinal consistency enforcement to track lesion changes reliably across time points. In addition, systematic follow-up to the Condition E finding is warranted before adopting adjacent-slice pseudo-RGB stacking in future ImageNet-pretrained 2D pipelines, for example by using a backbone pretrained without the implicit assumption of spatially identical input channels or by evaluating on datasets with thinner slice spacing where adjacent-slice anatomical change is smaller.

## 5. Conclusions

We have presented a 2D U-Net with a ResNet50 encoder that employs a pseudo-RGB FLAIR input encoding alongside a single-slice replication alternative (Condition E) for multiple sclerosis lesion segmentation. A two-phase transfer learning protocol and test-time augmentation complement the architecture. On the MSLesSeg2024 test set, the proposed pseudo-RGB model achieves a DSC of 0.7137 (95% CI: 0.6845–0.7194) and the lowest false negative pixel count per slice (FNV = 33.6 px/slice) among the FLAIR-only-or-fused evaluated configurations, achieving statistically comparable DSC to a patient-matched 3D nnU-Net baseline (p=0.68–0.79, not significant) while attaining a substantially higher pixel-level AUC (0.963 vs. 0.773). A supplementary ablation additionally shows that a simpler single-slice replication input (Condition E) significantly outperforms the pseudo-RGB encoding on this dataset (p<0.001), indicating that the anticipated inter-slice context benefit of pseudo-RGB stacking did not materialise here and warrants reconsideration in future ImageNet-pretrained 2D pipelines for this task (Section 4.1). These results demonstrate that a purpose-built lightweight 2D pipeline can match volumetrically aware 3D baselines’ segmentation overlap on constrained datasets at a fraction of the training and inference cost, with particular strength in pixel-level discrimination. Cross-dataset evaluation on MSSEG 2016 confirms generalisability across acquisition protocols: zero-shot DSC = 0.6562, recovering to DSC = 0.7046 after brief fine-tuning (30 epochs), which is within a single percentage point of in-domain performance. Thus, the proposed method provides a practical foundation for automated MS lesion quantification in routine clinical practice.

## Figures and Tables

**Table 1:** Ablation study on the MSLesSeg2024 test set (12 patients, approximately 1650 slices). Best value in each column is **bold**. FNV = mean false negative pixel count per slice (px/slice); HD95 in pixel space (224×224 images); ↑ = higher is better; ↓ = lower is better.

Cond.	Input	TTA	DSC ↑	IoU ↑	AUC ↑	HD95 ↓	LesF1 ↑	FNV ↓
A	Pseudo-RGB FLAIR	×	0.7133	0.6557	0.9754	24.34	0.6740	36.6
B	T1 + FLAIR	×	0.7017	0.6449	**0.9822**	26.30	0.6480	41.6
C	T1 + T2 + FLAIR	×	0.6910	0.6350	0.9743	25.43	0.6612	36.5
D (Ours)	Pseudo-RGB FLAIR	✓	0.7137	0.6571	0.9628	**23.39**	0.6771	**33.6**
E	Single-slice × 3	×	**0.7314**	**0.6739**	0.9628	—	**0.7075**	—

**Table 2:** Wilcoxon signed-rank test results for pairwise comparisons of per-slice DSC on the test set (n≈1650 slices). Significance threshold α=0.05; *** p<0.001; ** p<0.01; ns not significant. For the D-vs.-A comparison, TTA was applied to both conditions to isolate the TTA effect within the pseudo-RGB FLAIR pipeline (Section 2.10); tied and non-tied pair counts are reported for this comparison as a large fraction of per-slice DSC differences are exactly zero. Rank-biserial *r* is reported for the D-vs.-A and E-vs.-A comparisons only; effect sizes for the remaining pairs were not computed as those comparisons are secondary and their significance is unambiguous (p<0.001 and p=0.0016).

Comparison	*p*-Value	Significance	$r_{\mathrm{rb}}$	Tied/Total
B vs. A	<0.001	***	—	—
C vs. A	0.0016	**	—	—
C vs. B	0.1631	ns	—	—
D vs. A	0.1828	ns	0.821	937/1659
E vs. A	<0.001	***	—	—

**Table 3:** Comparison with 3D nnU-Net baseline on the MSLesSeg2024 test set. Both methods are trained on the identical 52-patient training set and evaluated on the identical 12-patient test set. HD95 is the true volumetric 95th-percentile Hausdorff distance in millimetres (voxel spacing 1 mm isotropic); small/medium/large DSC use identical maximum-axial cross-sectional area size strata for both methods (Section 2.9). ↑ = higher is better; ↓ = lower is better.

Method	DSC ↑	IoU ↑	AUC ↑	HD95 (mm) ↓
3D nnU-Net [10]	0.7263	0.5836	0.7726	10.65
95% CI	(0.6503–0.7912)	(0.4996–0.6608)	(0.7450–0.7963)	—
Proposed (2D, Cond. D)	0.7137	0.6571	**0.9628**	12.49
95% CI	(0.6845–0.7194)	(0.6272–0.6632)	(0.9422–0.9793)	—

**Table 4:** Cross-dataset generalisation on MSSEG 2016 (8 test patients, 1387 slices). Condition D (FLAIR-only pseudo-RGB with TTA) weights trained on MSLesSeg2024 are applied zero-shot and after 30-epoch fine-tuning. In-domain performance (MSLesSeg2024, Condition D) shown for reference—all rows use TTA at inference, so Condition D (not the no-TTA Condition A of Table 1) is the correct in-domain reference. TTA applied in all inference runs. FPV = false-positive pixels/slice; FNV = false-negative pixels/slice. † No large lesions (>1000 px) present in MSSEG 2016 test partition.

Setting	DSC	AUC	HD95	LesF1	FPV	FNV
MSLesSeg2024—in-domain (Cond D)	0.7137	0.9630	24.36	0.6756	—	34.3
MSSEG 2016—zero-shot	0.6562	0.9647	24.86	0.5943	6.6	66.9
MSSEG 2016—fine-tuned (30 ep)	**0.7046**	**0.9917**	**19.91**	**0.6529**	37.0	**27.6**

† Size-stratified DSC (small < 144 px/medium 144–1000 px): zero-shot 0.266/0.672; fine-tuned 0.338/0.795.

## Data Availability

The MSLesSeg2024 dataset is publicly available via the challenge at https://iplab.dmi.unict.it/mfs/ms-les-seg/ (accessed on 22 June 2026). Code and model weights for the proposed method will be made available upon acceptance.
